# BOLD Responses in Human Primary Visual Cortex are Insensitive to Substantial Changes in Neural Activity

**DOI:** 10.3389/fnhum.2013.00076

**Published:** 2013-03-11

**Authors:** J. B. Swettenham, S. D. Muthukumaraswamy, K. D. Singh

**Affiliations:** ^1^Oxford Centre for Human Brain Activity (OHBA), Department of Psychiatry, University of OxfordOxford, UK; ^2^Cardiff University Brain Research Imaging Centre (CUBRIC), School of Psychology, Cardiff UniversityCardiff, UK

**Keywords:** magnetoencephalography, functional magnetic resonance imaging, visual system, gamma oscillations, luminance, color

## Abstract

The relationship between blood oxygenation level dependent-functional magnetic resonance imaging (BOLD-fMRI) and magnetoencephalography (MEG) metrics were explored using low-level visual stimuli known to elicit a rich variety of neural responses. Stimuli were either perceptually isoluminant red/green or luminance-modulated black/yellow square-wave gratings with spatial frequencies of 0.5, 3, and 6 cycles per degree. Neural responses were measured with BOLD-fMRI (3-tesla) and whole head MEG. For all stimuli, the BOLD response showed bilateral activation of early visual cortex that was greater in the contralateral hemisphere. There was variation between individuals but weak, or no evidence, of amplitude dependence on either spatial frequency or the presence of luminance contrast. In contrast, beamformer analysis of MEG data showed activation in contralateral early visual cortex and revealed: (i) evoked responses with stimulus-dependent amplitude and latency; (ii) gamma and high-beta oscillations, with spatial frequency dependent peaks at approximately 30 and 50 Hz, but only for luminance-modulated gratings; (iii) The gamma and beta oscillations appeared to show different spatial frequency tuning profiles; (iv) much weaker gamma and beta responses, and at higher oscillation frequencies, for isoluminant compared to luminance-modulated gratings. The results provide further evidence that the relationship between the fMRI-BOLD response and cortical neural activity is complex, with BOLD-fMRI being insensitive to substantial changes in neural activity. All stimuli were clearly visible to participants and so the paucity of gamma oscillations to isoluminant stimuli is inconsistent with theories of their role in conscious visual perception.

## Introduction

Neuroimaging techniques such as magnetoencephalography (MEG) and functional magnetic resonance imaging (fMRI) provide enormous potential for the non-invasive study of human brain function. However, interpretation of neuroimaging data is limited by a lack of knowledge of how neural (and non-neural, e.g., glial) activity contributes to the signals being measured. To explore these relationships, results obtained with one neuroimaging technique can be compared with results from another neuroimaging technique, or to invasive recordings from animals. One such study in anesthetized macaque demonstrated that stimuli contrast positively correlated with the fMRI blood oxygenation level dependent (BOLD) response and with local field potentials (LFPs) measured simultaneously in primary visual cortex (Logothetis et al., 2001). Unlike multiunit activity, which reveals neuronal spiking activity, the LFP reflects local regional processing and Logothetis et al. concluded that the majority of the BOLD response reflects the high-energy demands of synaptic activity. Comparably, gamma frequency oscillations, measured non-invasively from human primary visual cortex using MEG, have also been shown to increase linearly with stimulus contrast (Hall et al., 2005), leading Hall et al. (2005) to postulate that the BOLD response and gamma oscillations may be coupled in some way, with each reflecting the other. Supporting this, a study in cat showed fluctuations of the hemodynamic response, as measured with optical imaging, correspond with fluctuations in the gamma response (Niessing et al., 2005).

In contrast, more recent multi-modal neuroimaging studies in human have found that, although BOLD and gamma band responses are closely co-localized spatially and show intrinsic temporal correlations (Mukamel et al., 2005; Niessing et al., 2005), the direct relationship between amplitudes of gamma oscillations and BOLD does not always hold when stimulus parameters such as spatial frequency are varied. For example, Muthukumaraswamy and Singh (2008, 2009) reported that MEG-measured gamma band amplitudes in primary visual cortex were substantially larger for gratings with a spatial frequency of 3 cycles per degree (cpd) compared with 0.5 cpd gratings, consistent with Adjamian et al. (2004), whereas BOLD responses were similar for the two spatial frequencies – although BOLD measures were sensitive to temporal frequency (Muthukumaraswamy and Singh, 2008) and contrast (Muthukumaraswamy and Singh, 2009). Thus, the relationship between BOLD responses and gamma oscillations, at least in visual cortex, is stimulus dependent.

Such inconsistencies in the relationship between different measures, within and between neuroimaging techniques, may provide critical information for increasing our understanding of neuronal processing (Singh, 2012). For this reason, we chose to use perceptually isoluminant red/green and luminance-modulated black/yellow gratings because they will primarily activate different cortical pathways, the parvo- and magno-cellular pathways respectively, and previous studies indicated they may elicit very different responses. For example, luminance-modulated gratings are well established as good inducers of gamma activity in both monkey (Friedman-Hill et al., 2000; Frien et al., 2000; Maldonado et al., 2000; Rols et al., 2001; Shapley et al., 2003; Gail et al., 2004; Henrie and Shapley, 2005; Belitski et al., 2008; Ray and Maunsell, 2010); and human (Hall et al., 2005; Hoogenboom et al., 2006; Hadjipapas et al., 2007; Adjamian et al., 2008; Muthukumaraswamy and Singh, 2008, 2009; Swettenham et al., 2009; Duncan et al., 2010) early visual cortex. However, similar isoluminant red/green gratings have previously been reported to both induce gamma activity in monkey (Rols et al., 2001) and human (Sannita et al., 2009) early visual cortex, and not to induce gamma activity in human (Adjamian et al., 2008). Adjamian et al. also noted decreases in beta oscillations in early visual cortex to chromatic stimuli, but beta increases in response to luminance-modulated stimuli. In addition, three spatial frequencies (0.5, 3, and 6 cpd) were chosen as gamma oscillations in human area V1 are optimally generated by high luminance contrast gratings at 3 cpd, but much less so by 0.5 and 6 cpd gratings (Adjamian et al., 2004; Muthukumaraswamy and Singh, 2009). Thus, we predicted both spatial frequency dependent and luminance dependent variations in neuronal responses. With fMRI, both types of stimuli have been shown to produce responses in visual cortex, including area V1 (for example, Kleinschmidt et al., 1996; Engel et al., 1997; Mullen et al., 2010).

## Materials and Methods

A total of 15 observers (eight males and seven females, aged 24–42 years) consented to participate in the study, which adhered to the tenets of the Declaration of Helsinki and was approved by the School of Psychology Ethics Committee, Cardiff University. Five observers took part in both the MEG and MRI experiments. Observers had normal or corrected-to-normal vision and no history of neurological dysfunction or injury. For all individuals, both MEG source localizations and fMRI-BOLD activation maps were co-registered to a previously acquired 1 mm isotropic resolution T1-weighted Fast Spoiled Gradient-Recalled Echo (FSPGR) anatomical scan.

### Stimuli

**Figure 1:**
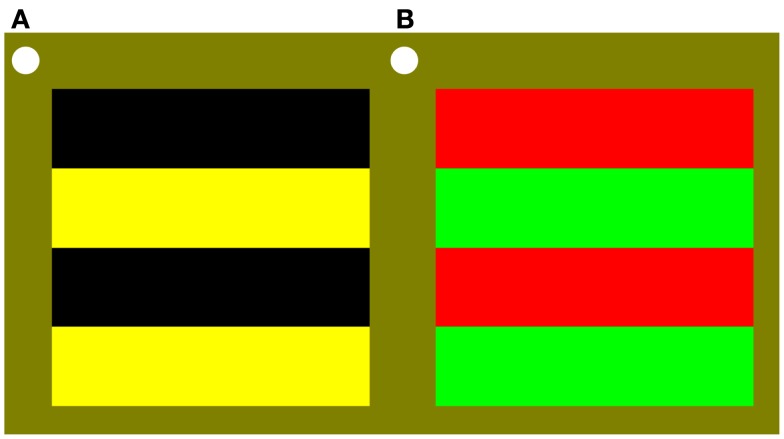
**Stimuli were 0.5 (as illustrated here), 3, or 6 cpd, horizontal, stationary, square-wave gratings (4° × 4°) that were either (A) black/yellow; or (B) perceptually isoluminant red/green**.

Stimuli were 0.5, 3, or 6 cpd, horizontal, stationary, square-wave gratings (4° × 4°) that were either black/yellow or perceptually isoluminant red/green (illustrated in Figure 1). For each observer, immediately before collecting data, red/green perceptual isoluminance was determined for each stimulus spatial frequency using the established method of minimum motion (Anstis and Cavanagh, 1983). Room lighting, participant position, grating size and location, were all the same as for data recording. The red luminance was 30 cd m^−2^ and the green luminance was set for each spatial frequency based on the results of the prior isoluminance testing. The yellow was the sum of the red and green colors such that the overall hue and luminance content of the black/yellow grating matched the red/green grating.

Stimuli were viewed binocularly in the lower right visual field with the top left corner of the grating close to fixation, as indicated by a continuously present white circle. Throughout the experiment the background was yellow with the same average hue content as the gratings and 30 cd m^−2^ luminance.

For the MEG experiment, there were 150 trials with 25 trials of each condition, and each trial was 7.5 s duration. For the fMRI experiment, there were 126 trials with 21 trials of each condition, and each trial was 12.0 s duration. With both MEG and fMRI, stimuli were presented for between 2.0 and 2.4 s, the duration being randomly selected by the program within 10 ms (i.e., one frame) bins. For the remaining trial time the screen showed the fixation circle on the average yellow background. Observers were instructed to maintain fixation and press a button when the grating disappeared.

### Magnetoencephalography

Ten observers (five males and five females, aged 25–41 years) took part in the MEG experiment. MEG data were recorded in a dimly lit, magnetically shielded room using a CTF 275-channel whole head radial gradiometer system in a single un-averaged run at a sampling rate of 1200 Hz. An additional 29 reference channels were recorded for noise cancelation purposes and the primary sensors were analyzed as synthetic third order gradiometers (Vrba and Robinson, 2001). Three of the 275 channels were turned off due to excessive sensor noise. Data collection took approximately 19 min per observer. The observer was fitted with three electromagnetic head coils which were localized relative to the MEG system immediately before and after the recording session. The mean head movement was 5 mm (SD 2 mm). Outside the shielded room, a three-dimensional digitizer (Fastrak; Polhemus, USA) was used to determine the position of these coils relative to the surface of the observer’s head. This head surface was matched to the observer’s own MR-defined head shape and these fiduciary locations could be verified using high-resolution digital photographs.

Stimuli were presented on a Mitsubishi Diamond Pro 2070 monitor and were controlled by a ViSaGe visual stimulus generator (Cambridge Research Systems, Kent, UK). The screen size was 1024 by 768 pixels and the monitor frame rate was 100 Hz. The monitor was outside the magnetically shielded room and viewed directly from within, at 2.1 m, through a cut-away portal in the shield.

For initial investigation of evoked responses, data was band-pass-filtered from 0.3 to 30 Hz and grand averages were computed for each condition. Evoked fields were localized using SAMerf (Robinson, 2004). A global covariance matrix of 0–100 Hz was calculated for each dataset and a corresponding set of beamformer weights computed. The evoked field was then computed, filtered from 1 to 40 Hz, passed through the beamformer weights and SAMerf images generated for 20 ms time bins from 40 to 200 ms.

Induced changes in oscillatory source power were localized using the synthetic aperture magnetometry (SAM) beamformer algorithm (Robinson and Vrba, 1999). Each dataset was band-pass-filtered using a fourth-order bi-directional IIR Butterworth filter into 10 Hz-wide frequency bands between 10 and 100 Hz. Evenly spaced frequency bands were used so that the accuracy of covariance matrix estimation would be equal for each frequency band (Brookes et al., 2008). The SAM algorithm was used to create differential images of source power (pseudo-*T* statistics) for 2 s of baseline (−2 to 0 s) compared to 2 s of visual stimulation (0 to 2 s). Time windows for baseline estimation were of equal duration to the time window of interest to achieve balanced covariance estimation. Details of the calculation of SAM pseudo-T source image statistics are described in detail in a number of sources (Vrba and Robinson, 2001; Cheyne et al., 2003; Singh et al., 2003; Hillebrand and Barnes, 2005; Hillebrand et al., 2005). For source localization, a multiple, local-spheres-forward model was derived by fitting spheres to the brain surface extracted by BET (Huang et al., 1999). Estimates of the three-dimensional distribution of source power were derived for each observer’s whole head at 3 mm isotropic voxel resolution. Note that eye blinks were not removed from the data because, in the unlikely event that blinks were time locked to the stimuli, the SAM spatial-filter will localize blink-related artifacts to the eyes (Bardouille et al., 2006).

For spatial locations of interest, activation time courses were calculated as if a sensor or an electrode were at that position, i.e., a virtual electrode. Time courses were constructed using SAM beamformer coefficients obtained using the individual condition covariance matrices band-pass-filtered between 0 and 100 Hz (Robinson and Vrba, 1999). For each of these virtual electrode time courses, time-frequency spectrograms were generated by determination of the time-varying amplitudes at each sample frequency. These envelopes were formed from the amplitude of the analytic signal derived using the Hilbert Transform between 1 and 100 Hz with 0.5 Hz step intervals and filtering with an 8 Hz-wide bandpass, third order Butterworth, filter (Le Van Quyen et al., 2001). The resulting spectrograms were either calculated separately for each trial and then averaged, in order to reveal both induced and evoked responses, or the spectrogram of the average across all trials was calculated to reveal the time-frequency content of the evoked responses. Here we present spectrograms as a percentage change from the mean baseline power at each frequency.

For group analysis, images were normalized into MNI (Montreal Neurological Institute) template space using an automated linear (affine) registration tool – FLIRT (Jenkinson and Smith, 2001). Non-parametric permutation tests were conducted using the full permutation set (1024) for each condition with 5 mm spatial smoothing of the inter-participant variance and thresholded using the omnibus test statistic value (obtained from the distribution of the largest activations in the whole volume, not each voxel, and so dealing with the multiple comparison problem) at *p* < 0.05 (Nichols and Holmes, 2002; Singh et al., 2003).

### Functional magnetic resonance imaging

Ten observers (six males and four females, aged 24–42 years) took part in the fMRI experiment.

MRI data were acquired on a 3-T General Electric HDx scanner using an eight-channel receive-only head RF coil (Medical Devices). Stimuli were controlled by a ViSaGe visual stimulus generator (Cambridge Research Systems, Kent, UK) and projected, via a Canon Xeed SX60 with Navitar SST300 zoom converter lens, down the rear bore of the scanner onto a screen behind the participant’s head. Observers viewed the screen via a front surfaced mirror mounted on the head coil. The viewing distance was 0.57 m, the screen size was 1024 by 768 pixels and the refresh rate was 60 Hz.

Functional magnetic resonance imaging data were acquired using a gradient echo EPI sequence, taking 30 slices of the whole brain, approximately in-line with the calcarine sulcus, at 3 mm isotropic voxel resolution with a 64 × 64 matrix size, echo time of 35 ms, 90° flip angle, and a TR of 2.0 s. Stimuli were presented over three runs, each approximately 8 min, with a short rest break for the observer between each run.

Analysis of fMRI data was performed using the FSL software library (www.fmrib.ox.ac.uk/fsl). The following pre-processing was applied: motion correction using MCFLIRT (Jenkinson et al., 2002), non-brain removal using Brain Extraction Tool (BET; Smith, 2002); spatial smoothing using a Gaussian kernel of full width half maxima (FWHM) 5 mm; mean-based intensity normalization (grand mean scaling) of all volumes by the same factor and high-pass temporal filtering (Gaussian-weighted least-squares straight line fitting, with sigma = 50 s). For each run, the General Linear Model (GLM) was used to model each of the six conditions using a 2 s on/10 s off boxcar to describe each stimulus. This boxcar function was convolved with a standard hemodynamic response function (HRF). To combine the three runs for each individual, a second-level analysis was performed using a fixed-effects analysis using FMRIBs Local Analysis of Mixed-Effects (FLAME; Beckmann et al., 2003; Woolrich et al., 2004). For each participant, the results of this analysis were co-registered with their previously acquired high-resolution anatomical scan. Group activation maps for each stimulus type were calculated by spatial normalization to a template brain using FLIRT (Jenkinson and Smith, 2001) and a mixed-effects analysis using FLAME to reveal significant activation clusters (*p* < 0.05 cluster level correction) for each stimulation condition.

## Results

In summary, luminance-modulated gratings elicited substantial increases in the magnitude of gamma frequency oscillations compared to baseline, but isoluminant red-green gratings did not. The BOLD responses did not show a simple relationship with any of the electrophysiological measures.

### Functional magnetic resonance imaging

**Figure 2:**
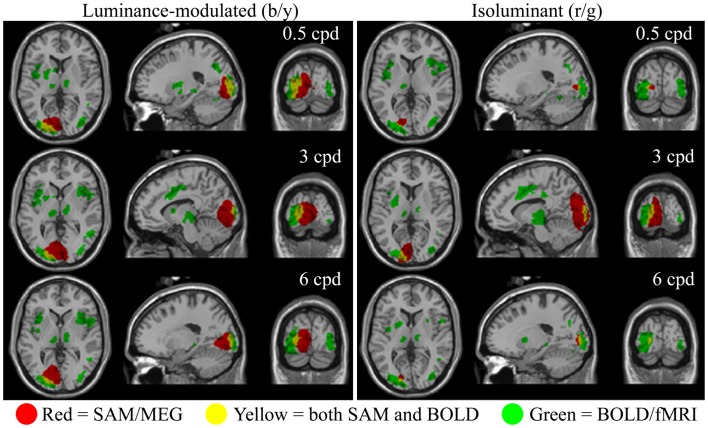
**Spatial localizations of group responses (*n* = 10) measured with BOLD/fMRI (green, mixed-effects, cluster level, *p* < 0.05), SAM/MEG (red, *p* < 0.05 corrected), and both (yellow) shown on the Montreal Neurological Institute (MNI) template brain**. Areas of MEG activity shown represent power increases during presentation of the grating stimuli (−2 to 0 vs. 0 to 2 s) in the frequency band with the largest response. This was in the gamma band in all cases (Lum-mod: 0.5 cpd: 50–60 Hz, *t* = 6.3, MNI: −19.1, −87.3, 3.0; 3 cpd: 50–60 Hz, *t* = 7.1, MNI: −1.0, −87.3, 3.0; 6 cpd: 50–60 Hz, *t* = 7.0, MNI: −15.1, −83.3, −3.0; Isolum: 0.5 cpd: 60–70 Hz, *t* = 4.9, MNI: −17.1, −83.3, 1.0; 3 cpd: 80–90 Hz, *t* = 6.8, MNI: −9.0, −93.4, 5.0; 6 cpd: 70–80 Hz, *t* = 4.9, MNI: −17.1, −87.3, 3.0).

The fMRI data showed differential BOLD responses to both isoluminant (red/green) and luminance-modulated (black/yellow) gratings at all spatial frequencies tested (0.5–6 cpd). Responses to each condition were consistently observed in contralateral early visual cortex. Figure 2 demonstrates that the locations of these responses were similar for all conditions. Less prominent areas of activation were also observed in the ipsilateral early visual cortex to all conditions.

**Figure 3:**
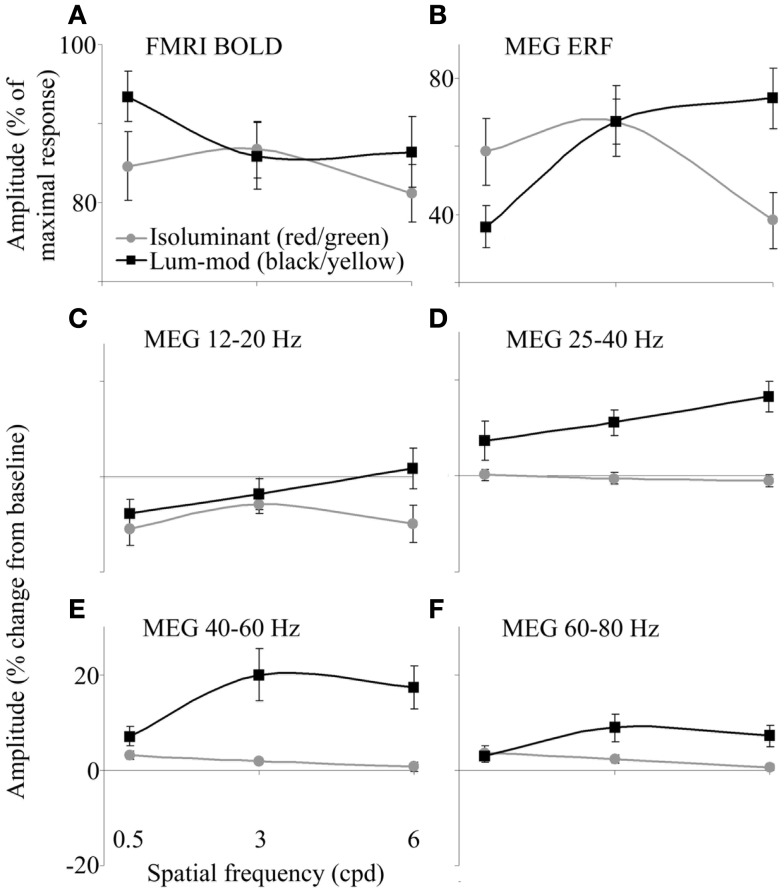
**(A)** fMRI-BOLD responses determined as a percentage of the largest response for each observer. **(B)** MEG ERF responses determined as a percentage of the largest response for each observer. **(C–F)** MEG-measured changes in spectral energy for four frequency bands in the 0.5–2 s time period compared with baseline. Error bars show the SEM.

Within the occipital cortex, the largest BOLD response for each participant was obtained and responses for each condition were calculated as a percentage of this maximum. Figure 3A shows the amplitude of the responses for each condition. There were no clear effects of the stimulus condition on the amplitude, or spatial extent, of BOLD activations [using SPSS to analyze amplitude values: main effect of color, *F*_(1,9)_ = 3.55, *p* = 0.092, *h*_P_ = 0.283; main effect of spatial frequency, *F*_(1,9)_ = 2.44, *p* = 0.144, *h*_P_ = 0.214 (Greenhouse–Geisser); interaction between color and frequency, *F*_(1,9)_ = 2.87, *p* = 0.083, *h*_P_ = 0.242].

### Magnetoencephalography

Synthetic aperture magnetometry analysis at the group level (*n* = 10) revealed that increases in gamma frequency power were occurring in response to isoluminant gratings, despite this rarely reaching significance at the individual level (see below). Figure 2 shows areas of increased gamma power in the peak frequency band superimposed on the normalized brain. The spatial extent of the activation associated with 3 cpd isoluminant gratings was comparable with that for luminance-modulated gratings, but the areas of activation for 0.5 and 6 cpd isoluminant gratings were much smaller. There was overlap between the areas of activation determined by the fMRI-BOLD response and the MEG response, although the MEG activation tended to be more medial and deeper. Note that the largest power changes in response to luminance-modulated gratings were in the 50–60 Hz band for all spatial frequencies, whereas the largest power changes to isoluminant gratings were in higher frequency bands (60–70, 80–90, and 70–80 Hz for 0.5, 3, and 6 cpd respectively).

Initial viewing of the averaged data, by overlaying all of the 272 sensor channels, showed there were prominent evoked responses, most often composed of two main peaks. These evoked responses were investigated using SAMerf, a beamformer that is optimized for localization of transient evoked responses (Robinson, 2004). SAMerf was performed in 20 ms time bins from 40 to 200 ms.

**Figure 4:**
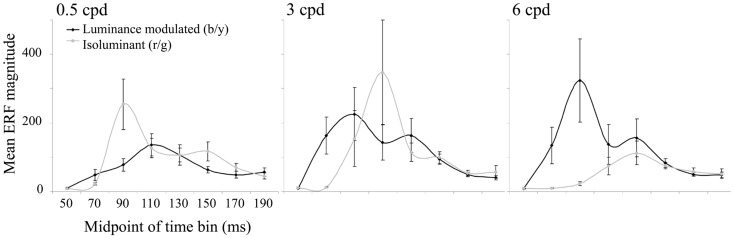
**The mean (*n* = 10) of the largest ERF in each 20 ms time bin from 40 to 200 ms is plotted for each condition**. Error bars show the SEM. Units are arbitrary.

The largest evoked responses were in early visual areas of the contralateral (left) hemisphere and locations were remarkably stable within an individual, both across conditions and across time windows. The curves plotted in Figure 4 show the magnitude of the largest evoked response in each time bin averaged over the 10 observers. For luminance-modulated black/yellow gratings at 0.5 cpd there is only one peak in amplitude (in the 100–120 ms time bin), whereas with 3 and 6 cpd gratings there are two peaks and a shift in dominance to the earlier peak (80–100 ms) with increasing spatial frequency can be clearly seen. For isoluminant red/green gratings the time bin with the largest magnitude shifted from 80–100 to 100–120 to 120–140 ms as spatial frequency increased from 0.5 to 3 to 6 cpd.

**Figure 5:**
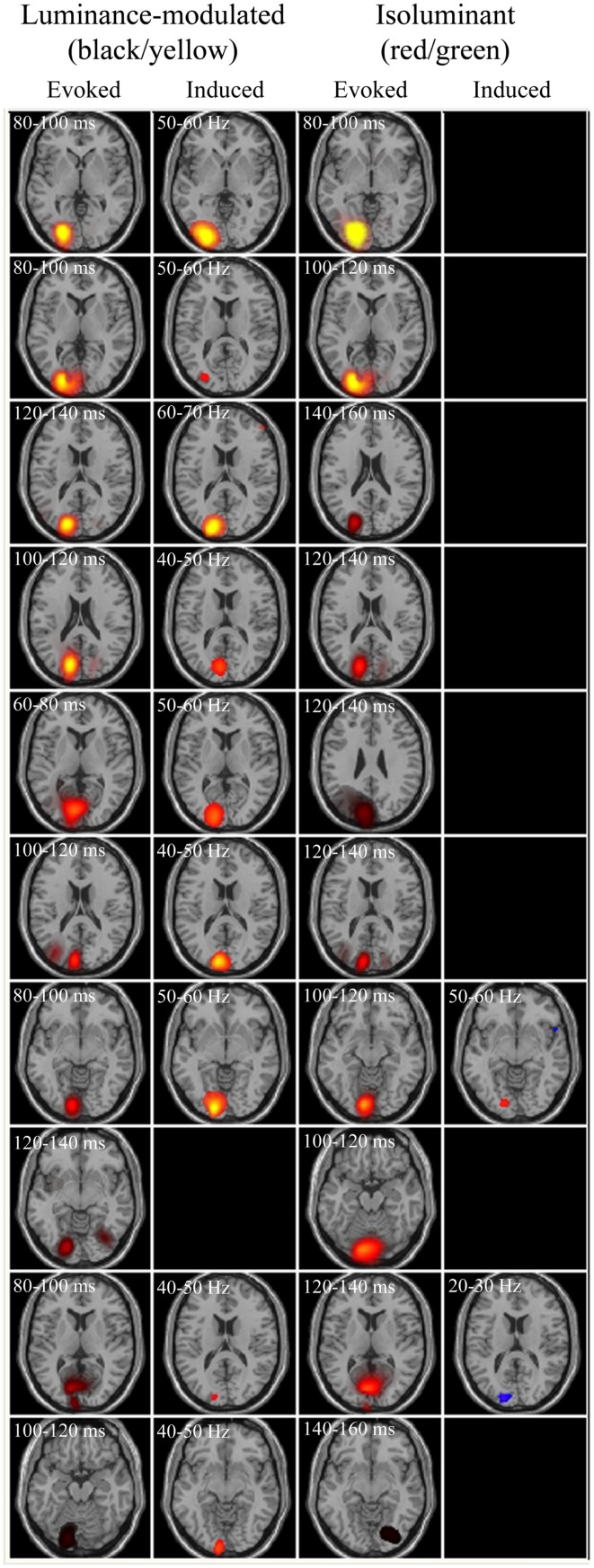
**Evoked and induced responses for each observer to luminance-modulated and isoluminant stimuli at 3 cpd**. Axial slices are through the peak activations. Each row represents an observer, with observers ranked in order of the largest evoked responses. Blue/purple colors depict energy decreases and orange/yellow colors depict energy increases. All evoked panels have an arbitrary color range of 500–5. Colors in the induced panels indicate the amplitude of the pseudo-*T* statistic (2 ≤ *T* ≤ 6).

For comparison with the evoked responses, power changes that may not be phase-locked to the stimuli (i.e., induced) were spatially localized using SAM, with nine non-overlapping frequency bands of 10 Hz width between 10 and 100 Hz and over 2 s time windows (pre- vs. post-grating onset). Results revealed that 3 cpd luminance-modulated gratings were the most effective at eliciting power changes, with 9 out of 10 observers having peak power changes with pseudo-*T* values >2 in at least one of the frequency bands (compared with 6/10 and 8/10 for 0.5 and 6 cpd luminance-modulated and 4/10, 2/10, and 3/10 for 0.5, 3, and 6 cpd isoluminant gratings respectively). Figure 5 shows evoked and induced responses to 3 cpd gratings for all observers. It can be seen that within an individual, the locations of the changes in evoked and induced power were similar and that the isoluminant red/green gratings did not produce large induced power changes. The coordinates of the peak SAM response to 3 cpd luminance-modulated gratings were compared with the coordinates of the largest evoked response for each observer (except the observer with no SAM peak) and the mean separation of the peaks was 0.87 cm (SD 0.35 cm) with no consistent direction to the separation.

One of the observers did not produce any measurable power changes (pseudo-*T* > 2) in any of the conditions or frequency bands, whilst for the remaining nine observers the most prominent finding was that luminance-modulated (black/yellow) gratings elicited increases in gamma frequency power in early visual areas of the contralateral hemisphere. Four observers showed SAM measured power changes in response to isoluminant gratings, these peaks also occurred in early visual areas of the left hemisphere and were generally smaller than the power changes to luminance-modulated gratings. For three out of four observers the largest changes to isoluminant gratings were power reductions in the 10–20 or 20–30 Hz frequency bands, whilst only one observer showed an increase in gamma frequency oscillations.

**Figure 6:**
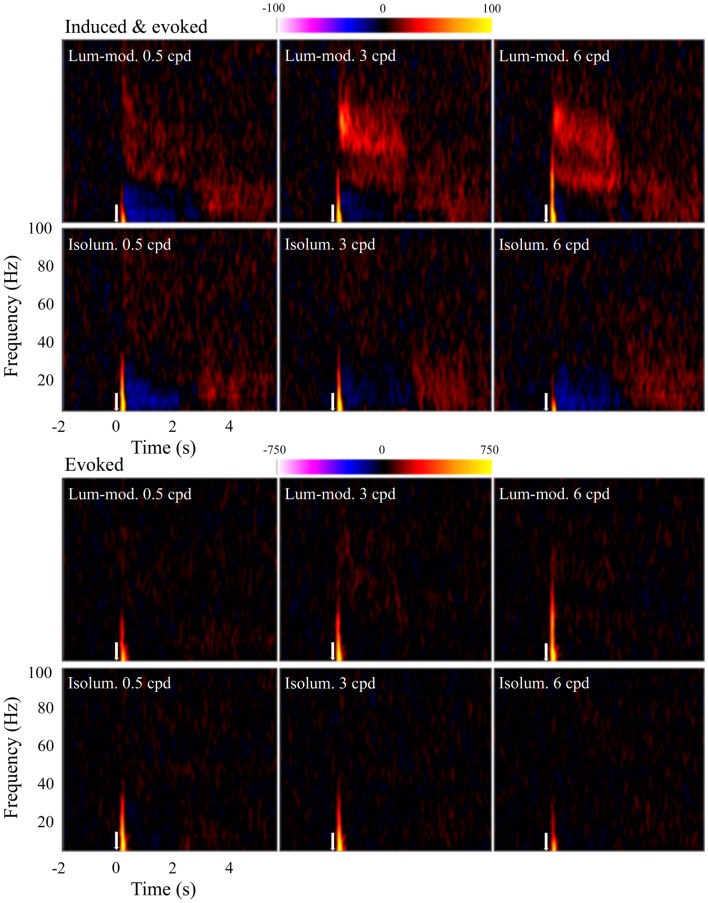
**Mean (*n* = 10) time-frequency plots showing activity at peak locations in contralateral, early visual cortex**. Top panels show both evoked and induced activity whereas lower panels show only evoked activity. Colors represent energy changes from baseline. Blue/purple colors depict energy decreases and orange/yellow colors depict energy increases. Stimulus onset was at time 0 s (white arrow) and continued until 2.0–2.4 s.

Time-frequency plots were calculated for observers at the locations of their largest evoked (SAMerf) response in each condition (*n* = 10) and their peak induced (SAM) response to 3 cpd luminance-modulated gratings (*n* = 9). These plots were qualitatively the same and so all plots shown are based on the SAMerf locations. Figure 6 shows the group (*n* = 10) time-frequency plots for each condition showing both induced and evoked activity (top panels) and just evoked activity (bottom panels). All plots show an initial, transient increase in energy relative to the baseline. As this component is seen in the bottom panels it indicates that this response is phase-locked and it reflects the tuning properties shown in Figure 3B. Only the top panels show other components, which can be inferred to be induced, i.e., non-phase-locked. Most strikingly, these induced components include a sustained increase in amplitude at gamma frequencies, particularly at 3 and 6 cpd, for luminance-modulated but not isoluminant gratings. In addition, for most conditions there is a decrease in amplitude at alpha and low-beta frequencies during stimulus presentation (0 to 2.0–2.4 s) that is followed by an increase in energy at stimulus offset.

**Figure 7:**
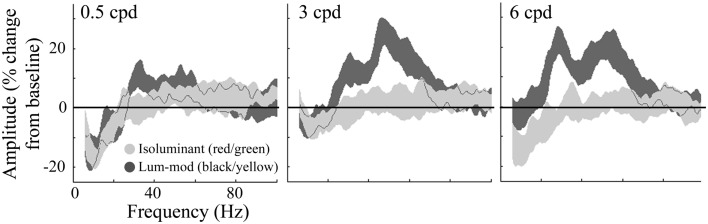
**Group average (*n* = 10) responses depicting the average power spectrum, over the 0.5–2 s time period, at virtual electrode positions in early visual cortex**. Responses to isoluminant red/green gratings are shown in light gray and responses to luminance-modulated gratings are shown in dark gray. The shaded regions indicate the SEM across all participants. The amplitude spectra, plotted as percentage change from the pre-stimulus baseline, are shown for each of the stimulus spatial frequencies. Note the increases in amplitude to luminance-modulated gratings occurred with two peaks, one at approximately 30 and another at approximately 50 Hz.

For the same virtual electrode positions, Figure 7 shows the shape and magnitude of the power spectra averaged over the 0.5–2 s time period. This time window was chosen to explore the sustained response and avoid contamination from the initial transient response. These plots again show an increase in power at gamma frequencies for all spatial frequencies of luminance-modulated, but not isoluminant, gratings. Whilst the gamma power increase associated with 0.5 cpd gratings was modest, the increases with 3 and 6 cpd gratings were larger and showed two distinct frequency peaks. The lower frequency power increase occurred with a peak at approximately 30 Hz, in the high-beta/low-gamma range, and the higher frequency power increase had a peak at approximately 50 Hz. Based on these spectra, four frequency bands were chosen for further temporal analysis. The first investigated the decrease in low-beta power (12–20 Hz), the second the high-beta/low-gamma frequency increase (25–40 Hz), the third the higher gamma frequency increase (40–60 Hz), and the fourth higher gamma frequencies at which the response was weaker (60–80 Hz).

**Figure 8:**
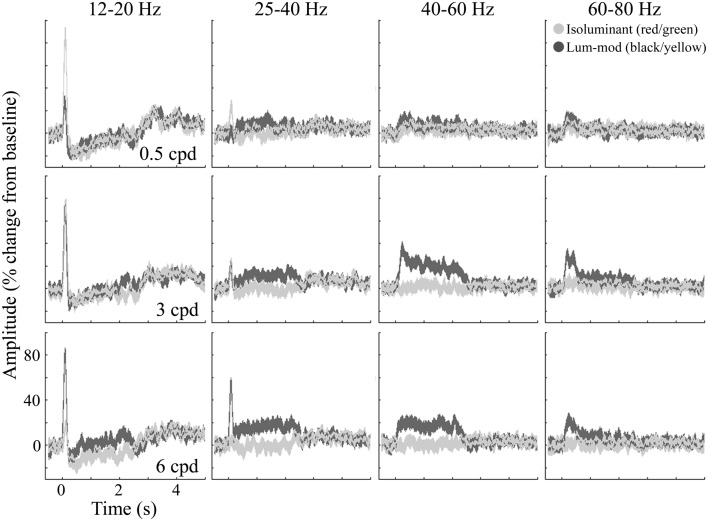
**Group average (*n* = 10) responses depicting the temporal evolution of the cortical response at virtual electrode positions in early visual cortex**. Responses to isoluminant red/green gratings are shown in light gray and responses to luminance-modulated gratings are shown in dark gray. The shaded regions indicate the SEM across all participants. The amplitude spectra, plotted as percentage change from the pre-stimulus baseline, are shown for each of the stimulus spatial frequencies and for a range of frequency bands chosen to illustrate the main effects found.

Figure 8 shows the temporal evolution of activity for these four frequency bands. At low-beta frequencies (12–20 Hz, first column), the response is characterized by an initial evoked power increase that is followed (with the exception of 6 cpd luminance-modulated gratings) by an induced decrease sustained for the stimulus presentation (2–2.4 s). After stimulus offset there is a rebound increase in low-beta power. At the high-beta/low-gamma frequency (25–40 Hz, second column), there is still some evidence of the evoked response. This is followed by a sustained power increase for 3 and 6 cpd luminance-modulated gratings that is larger for 6 cpd gratings. Similarly, at 40–60 Hz (third column), there is a sustained power increase for 3 and 6 cpd luminance-modulated gratings. For 3 cpd gratings, this increase is greater than the increase in the 25–40 Hz band and shows some adaptation over the first 500 ms. These figures demonstrate evidence of a difference in spatial frequency tuning for the beta and gamma responses. In comparison, at 60–80 Hz (fourth column) there is little evidence of a sustained increase in power but for luminance-modulated 3 and 6 cpd gratings there is a transient increase in power for approximately the first 500 ms.

In order to summarize the MEG responses for each condition, tuning curves are shown in Figures 3B–F. Figure 3B shows the magnitude of the evoked peaks. The magnitudes of the MRI responses and that of the evoked peaks were not correlated (for example, for black/yellow gratings: *r* = −0.44, *p* > 0.05; for red/green gratings: *r* = 0.06, *p* > 0.05).

The amplitudes of energy changes, averaged both over the 0.5–2 s time window and over the four frequency bands, are shown in Figures 3C–F. The absence of a reduction in 12–20 Hz power for luminance-modulated 6 cpd gratings (Figure 3C) is clearly illustrated. Also, at 40–60 (Figure 3E) and 60–80 Hz (Figure 3F), a slight increase in power is shown for isoluminant gratings with low spatial frequencies (0.5 and 3 cpd). At 0.5 cpd spatial frequency, this increase is comparable for isoluminant and luminance-modulated gratings.

## Discussion

This study demonstrates that the two neuroimaging techniques, MEG and BOLD-fMRI, provide complementary but not interchangeable data on brain activity. In early visual cortex, MEG revealed substantial differences in oscillatory activity in response to different stimuli types (Figures 3 and 6), whereas BOLD responses were similar for all stimuli (Figures 2 and 3). Thus, at least in human primary visual cortex, BOLD responses are independent from gamma oscillations, a finding we have previously shown with responses to varying spatial frequency (Muthukumaraswamy and Singh, 2008, 2009).

The similarity of our BOLD responses across stimuli types may have been, at least in part, a consequence of the low temporal resolution (TR = 2 s) we used. Such temporal resolutions, or lower, are common for human fMRI studies where the whole brain is scanned. However, Goense and Logothetis (2008) used much shorter repetition times (250 ms) to measure BOLD responses localized at electrode sites in monkeys, and reported more sensitivity in the correlation of the BOLD with specific frequency bands.

Luminance-modulated and isoluminant stimuli were chosen because they would primarily, although not exclusively, activate different visual pathways – magno- and parvo-cellular for luminance-modulated and isoluminant stimuli respectively. Strikingly, the sustained increases in gamma frequency oscillations in early visual cortex, which were prominent in response to luminance-modulated gratings, were far weaker to isoluminant gratings. This indicates that luminance contours may play a key role in the generation of gamma oscillations and is in agreement with the findings that stimulus-driven gamma response amplitude is proportional to luminance contrast (Hall et al., 2005) and that gamma responses are not elicited by chromatic gratings (Adjamian et al., 2008; but, see Sannita et al., 2009). Both stimuli types generated comparable regions of BOLD activity, consistent with several studies that have reported robust BOLD responses to color stimuli in primary visual cortex (Kleinschmidt et al., 1996; Engel et al., 1997; Mullen et al., 2010). Our results suggest that the pathway by which input has arrived in the cortex has a profound influence on the generation of gamma frequency oscillations, but not on BOLD responses.

We examined the relationships between the BOLD responses and evoked and induced MEG measures of neural activity. Our conclusion is that, despite the richness of MEG data, there was not a simple relationship between any one measure and the BOLD response. Whether changes in oscillatory activity increase, decrease, or do not effect, brain metabolism may depend on the oscillatory frequencies and the brain areas involved, and the BOLD response may reflect the combined effects of many phase- and non-phase-locked electrophysiological responses (Singh, 2012). A previous study found a negative correlation between oscillations lower than 45 Hz and the BOLD response, and a positive correlation between higher frequency oscillations and the BOLD response (Zumer et al., 2010). Although we do not have sufficient data to explore this properly it would seem that this general observation is consistent with our main findings for the luminance-modulated gratings (see Figure 6). However, the BOLD responses were comparable with those in response to the isoluminant gratings which did not elicit pronounced oscillations. BOLD responses were obtained to each stimuli type, so BOLD is sensitive to neural changes, but as the BOLD is the result of a complex mixture of neural responses reduced to metabolic activity, it is clear from this study that the BOLD response can be insensitive to large differences in oscillatory activity.

There are many methods for analyzing MEG data, each with their own sensitivities. Here, we used two types of beamformer analysis, one designed for identifying evoked responses (SAMerf; Robinson, 2004), and one for non-phase-locked responses (SAM; Robinson and Vrba, 1999). We looked at responses at the group level as well as the individual level. The locations of the evoked peak responses for individuals were highly consistent across conditions, being in contralateral early visual cortex. Within individuals these locations were then very similar to the peak locations of the induced, non-phase-locked, responses. However, not all conditions elicited induced activations, in particular the isoluminant conditions were not effective at producing gamma or high-beta oscillations (as mentioned above). In contrast, when responses were examined at the group level, gamma oscillations were observed to isoluminant stimuli, particularly at 3 cpd. Such discordant results have been attributed to the inhomogeneous smoothness of MEG data, in that signals of high strength have narrow FWHM, and signals of lower strength have wider FWHM, i.e., more spread (Barnes et al., 2004). The consequence of this spatial inhomogeneity, combined with individual variations in brain structure, is that strong, highly spatially localized, signals within individuals may not align at the group level and so are missed or reduced; conversely, weaker, widely spread, signals are more likely to overlap across individuals and produce a group response.

Peak gamma oscillation frequencies were higher for the red/green stimuli than for the luminance-modulated gratings, as observed with the group level activations, and for each of the spatial frequencies (Figure 2). Also, the induced oscillatory responses to luminance-modulated gratings occurred with two frequency peaks, one in the high-beta/low-gamma (∼30 Hz) and one in the higher gamma range (∼50 Hz; see Figure 7). There have been several explanations put forward to explain what influences the frequency of gamma oscillations. One influence, directly relevant to our stimuli, is the spatial frequency of visual stimuli. In our data, the high-beta/low-gamma peak, at approximately 30 Hz, became stronger as stimulus spatial frequency increased from 3 to 6 cpd, consistent with the findings of Hadjipapas et al. (2007). There is also evidence of two peaks in a similar MEG study using black/white gratings (Figure 4; Swettenham et al., 2009). In addition to spatial frequency, other stimulus properties, such as orientation (Duncan et al., 2010), and motion (Swettenham et al., 2009), have also been shown to affect the spectral properties of the gamma response in human early visual cortex. All of our stimuli were clearly visible and so our findings do not support a previous human MEG study that claimed that low-gamma frequencies reflect conscious visual perception and higher gamma frequencies reflect spatial attention (Wyart and Tallon-Baudry, 2008). However, although Wyart and Tallon-Baudry claim their gamma sources were from occipital cortex, this was based on scalp topographies and their findings could relate to higher visual, or other neighboring, cortical areas.

Many electrophysiological studies have also aimed to understand the basis of oscillation frequency. A recent electrophysiology study on anesthetized cat found that gamma oscillation frequency in primary visual cortex was tuned for the drifting direction of a grating, with more optimal stimuli inducing higher oscillation frequencies (Feng et al., 2010). Higher gamma frequencies in response to higher luminance contrast have also recently been reported in primary visual cortex of awake macaque, an effect that could not be sufficiently attributed to attention (Ray and Maunsell, 2010). In addition, very high gamma activity, beyond that observed in this study (>80 Hz), may not be a true rhythm at all and may simply reflect neuronal firing rate (Ray and Maunsell, 2011). Using tissue from various brain areas, *in vitro* studies have demonstrated that the two different frequency oscillations may derive from specific cortical layers, be independent and have different pharmacological profiles (Oke et al., 2010). Unfortunately, we are not able to associate cortical layers with the magno- or parvo-cellular stimuli, as upon arrival in primary visual cortex the pathways become mixed and there are many interactions between cortical layers (Lennie et al., 1990; De Valois et al., 2000; Johnson et al., 2001, 2004; Wachtler et al., 2003; Solomon and Lennie, 2005; Conway and Livingstone, 2006; Horwitz et al., 2007).

In conclusion, both MRI and MEG demonstrated responses to all the visual stimuli used here. There was general spatial agreement between the peak locations of evoked responses as measured with MEG, and the peak BOLD responses as measured with fMRI. However, the magnitude of the BOLD responses were comparable across conditions and did not reflect the substantial oscillatory differences between conditions as observed with MEG.

## Conflict of Interest Statement

The authors declare that the research was conducted in the absence of any commercial or financial relationships that could be construed as a potential conflict of interest.

## References

[B1] Adjamian P. Hadjipapas A. Barnes G. R. Hillebrand A. Holliday I. E. (2008). Induced gamma activity in primary visual cortex is related to luminance and not color contrast: an MEG study. J. Vis. 8, 1–7 10.1167/8.5.1 19146237

[B2] Adjamian P. Holliday I. E. Barnes G. R. Hillebrand A. Hadjipapas A. Singh K. D. (2004). Induced visual illusions and gamma oscillations in human primary visual cortex. Eur. J. Neurosci. 20, 587–592 10.1111/j.1460-9568.2004.03495.x 15233769

[B3] Anstis S. Cavanagh P. (1983). “A minimum motion technique for judging equiluminance,” in Colour Vision: Psychophysics and Physiology, ed. Mollon J. (London: Academic Press), 155–166

[B4] Bardouille T. Picton T. W. Ross B. (2006). Correlates of eye blinking as determined by synthetic aperture magnetometry. Clin. Neurophysiol. 117, 952–958 10.1016/j.clinph.2006.01.021 16564205

[B5] Barnes G. R. Hillebrand A. Fawcett I. P. Singh K. D. (2004). Realistic spatial sampling for MEG beamformer images. Hum. Brain Mapp. 23, 120–127 10.1002/hbm.20047 15340934 PMC6872013

[B6] Beckmann C. F. Jenkinson M. Smith S. M. (2003). General multilevel linear modeling for group analysis in FMRI. Neuroimage 20, 1052–1063 10.1016/S1053-8119(03)00435-X 14568475

[B7] Belitski A. Gretton A. Magri C. Murayama Y. Montemurro M. A. Logothetis N. K. (2008). Low-frequency local field potentials and spikes in primary visual cortex convey independent visual information. J. Neurosci. 28, 5696–5709 10.1523/JNEUROSCI.0009-08.2008 18509031 PMC6670798

[B8] Brookes M. J. Vrba J. Robinson S. E. Stevenson C. M. Peters A. M. Barnes G. R. (2008). Optimising experimental design for MEG beamformer imaging. Neuroimage 39, 1788–1802 10.1016/j.neuroimage.2007.09.050 18155612

[B9] Cheyne D. Gaetz W. Garnero L. Lachaux J. P. Ducorps A. Schwartz D. (2003). Neuromagnetic imaging of cortical oscillations accompanying tactile stimulation. Brain Res. Cogn. Brain Res. 17, 599–611 10.1016/S0926-6410(03)00173-3 14561448

[B10] Conway B. R. Livingstone M. S. (2006). Spatial and temporal properties of cone signals in alert macaque primary visual cortex. J. Neurosci. 26, 10826–10846 10.1523/JNEUROSCI.2091-06.2006 17050721 PMC2963176

[B11] De Valois R. L. Cottaris N. P. Elfar S. D. Mahon L. E. Wilson J. A. (2000). Some transformations of color information from lateral geniculate nucleus to striate cortex. Proc. Natl. Acad. Sci. U.S.A. 97, 4997–5002 10.1073/pnas.97.1.512 10781111 PMC18346

[B12] Duncan K. K. Hadjipapas A. Li S. Kourtzi Z. Bagshaw A. Barnes G. (2010). Identifying spatially overlapping local cortical networks with MEG. Hum. Brain Mapp. 31, 1003–1016 10.1002/hbm.20912 19998365 PMC3179596

[B13] Engel S. Zhang X. M. Wandell B. (1997). Colour tuning in human visual cortex measured with functional magnetic resonance imaging. Nature 388, 68–71 10.1038/40398 9214503

[B14] Feng W. Havenith M. N. Wang P. Singer W. Nikolic D. (2010). Frequencies of gamma/beta oscillations are stably tuned to stimulus properties. Neuroreport 21, 680–684 20495495 10.1097/WNR.0b013e32833ae9d1

[B15] Friedman-Hill S. Maldonado P. E. Gray C. M. (2000). Dynamics of striate cortical activity in the alert macaque: I. Incidence and stimulus-dependence of gamma-band neuronal oscillations. Cereb. Cortex 10, 1105–1116 10.1093/cercor/10.11.1105 11053231

[B16] Frien A. Eckhorn R. Bauer R. Woelbern T. Gabriel A. (2000). Fast oscillations display sharper orientation tuning than slower components of the same recordings in striate cortex of the awake monkey. Eur. J. Neurosci. 12, 1453–1465 10.1046/j.1460-9568.2000.00025.x 10762373

[B17] Gail A. Brinksmeyer H. J. Eckhorn R. (2004). Perception-related modulations of local field potential power and coherence in primary visual cortex of awake monkey during binocular rivalry. Cereb. Cortex 14, 300–313 10.1093/cercor/bhg129 14754869

[B18] Goense J. B. M. Logothetis N. K. (2008). Neurophysiology of the BOLD fMRI signal in awake monkeys. Curr. Biol. 18, 631–640 10.1016/j.cub.2008.03.054 18439825

[B19] Hadjipapas A. Adjamian P. Swettenham J. B. Holliday I. E. Barnes G. R. (2007). Stimuli of varying spatial scale induce gamma activity with distinct temporal characteristics in human visual cortex. Neuroimage 35, 518–530 10.1016/j.neuroimage.2007.01.002 17306988

[B20] Hall S. D. Holliday I. E. Hillebrand A. Singh K. D. Furlong P. L. Hadjipapas A. (2005). The missing link: analogous human and primate cortical gamma oscillations. Neuroimage 26, 13–17 10.1016/j.neuroimage.2005.01.009 15862200

[B21] Henrie J. A. Shapley R. (2005). LFP power spectra in V1 cortex: the graded effect of stimulus contrast. J. Neurophysiol. 94, 479–490 10.1152/jn.00919.2004 15703230

[B22] Hillebrand A. Barnes G. R. (2005). Beamformer analysis of MEG data. Int. Rev. Neurobiol. 68, 149–171 10.1016/S0074-7742(05)68006-3 16443013

[B23] Hillebrand A. Singh K. D. Holliday I. E. Furlong P. L. Barnes G. R. (2005). A new approach to neuroimaging with magnetoencephalography. Hum. Brain Mapp. 25, 199–211 10.1002/hbm.20102 15846771 PMC6871673

[B24] Hoogenboom N. Schoffelen J. M. Oostenveld R. Parkes L. M. Fries P. (2006). Localizing human visual gamma-band activity in frequency, time and space. Neuroimage 29, 764–773 10.1016/j.neuroimage.2005.08.043 16216533

[B25] Horwitz G. D. Chichilnisky E. J. Albright T. D. (2007). Cone inputs to simple and complex cells in V1 of awake macaque. J. Neurophysiol. 97, 3070–3081 10.1152/jn.00965.2006 17303812

[B26] Huang M. X. Mosher J. C. Leahy R. M. (1999). A sensor-weighted overlapping-sphere head model and exhaustive head model comparison for MEG. Phys. Med. Biol. 44, 423–440 10.1088/0031-9155/44/2/010 10070792

[B27] Jenkinson M. Bannister P. Brady M. Smith S. (2002). Improved optimization for the robust and accurate linear registration and motion correction of brain images. Neuroimage 17, 825–841 10.1006/nimg.2002.1132 12377157

[B28] Jenkinson M. Smith S. (2001). A global optimisation method for robust affine registration of brain images. Med. Image Anal. 5, 143–156 10.1016/S1361-8415(01)00036-6 11516708

[B29] Johnson E. N. Hawken M. J. Shapley R. (2001). The spatial transformation of color in the primary visual cortex of the macaque monkey. Nat. Neurosci. 4, 409–416 10.1038/86061 11276232

[B30] Johnson E. N. Hawken M. J. Shapley R. (2004). Cone inputs in macaque primary visual cortex. J. Neurophysiol. 91, 2501–2514 10.1152/jn.01043.2003 14749310

[B31] Kleinschmidt A. Lee B. B. Requardt M. Frahm J. (1996). Functional mapping of color processing by magnetic resonance imaging of responses to selective P- and M-pathway stimulation. Exp. Brain Res. 110, 279–288 10.1007/BF00228558 8836691

[B32] Le Van Quyen M. Foucher J. Lachaux J. P. Rodriguez E. Lutz A. Martinerie J. (2001). Comparison of Hilbert transform and wavelet methods for the analysis of neuronal synchrony. J. Neurosci. Methods 111, 83–98 10.1016/S0165-0270(01)00372-7 11595276

[B33] Lennie P. Krauskopf J. Sclar G. (1990). Chromatic mechanisms in striate cortex of macaque. J. Neurosci. 10, 649–669 2303866 10.1523/JNEUROSCI.10-02-00649.1990 PMC6570166

[B34] Logothetis N. K. Pauls J. Augath M. Trinath T. Oeltermann A. (2001). Neurophysiological investigation of the basis of the fMRI signal. Nature 412, 150–157 10.1038/35084005 11449264

[B35] Maldonado P. E. Friedman-Hill S. Gray C. M. (2000). Dynamics of striate cortical activity in the alert macaque: II. Fast time scale synchronization. Cereb. Cortex 10, 1117–1131 10.1093/cercor/10.11.1117 11053232

[B36] Mukamel R. Gelbard H. Arieli A. Hasson U. Fried I. Malach R. (2005). Coupling between neuronal firing, field potentials, and fMR1 in human auditory cortex. Science 309, 951–954 10.1126/science.1110913 16081741

[B37] Mullen K. T. Thompson B. Hess R. F. (2010). Responses of the human visual cortex and LGN to achromatic and chromatic temporal modulations: an fMRI study. J. Vis. 10, 13 10.1167/10.10.13 21106678

[B38] Muthukumaraswamy S. D. Singh K. D. (2008). Spatiotemporal frequency tuning of BOLD and gamma band MEG responses compared in primary visual cortex. Neuroimage 40, 1552–1560 10.1016/j.neuroimage.2008.01.052 18337125

[B39] Muthukumaraswamy S. D. Singh K. D. (2009). Functional decoupling of BOLD and gamma-band amplitudes in human primary visual cortex. Hum. Brain Mapp. 30, 2000–2007 10.1002/hbm.20644 18729078 PMC6870698

[B40] Nichols T. E. Holmes A. P. (2002). Nonparametric permutation tests for functional neuroimaging: a primer with examples. Hum. Brain Mapp. 15, 1–25 10.1002/hbm.1058 11747097 PMC6871862

[B41] Niessing J. Ebisch B. Schmidt K. E. Niessing M. Singer W. Galuske R. A. W. (2005). Hemodynamic signals correlate tightly with synchronized gamma oscillations. Science 309, 948–951 10.1126/science.1110948 16081740

[B42] Oke O. O. Magony A. Anver H. Ward P. D. Jiruska P. Jefferys J. G. R. (2010). High-frequency gamma oscillations coexist with low-frequency gamma oscillations in the rat visual cortex in vitro. Eur. J. Neurosci. 31, 1435–1445 10.1111/j.1460-9568.2010.07171.x 20384769

[B43] Ray S. Maunsell J. H. R. (2010). Differences in gamma frequencies across visual cortex restrict their possible use in computation. Neuron 67, 885–896 10.1016/j.neuron.2010.08.004 20826318 PMC3001273

[B44] Ray S. Maunsell J. H. R. (2011). Different origins of gamma rhythm and high-gamma activity in macaque visual cortex. PLoS Biol. 9:e1000610 10.1371/journal.pbio.1000610 21532743 PMC3075230

[B45] Robinson S. E. (2004). Localization of event-related activity by SAM(erf). Neurol. Clin. Neurophysiol. [Epub ahead of print]. 16012649

[B46] Robinson S. E. Vrba J. (1999). “Functional neuroimaging by synthetic aperture magnetometry (SAM),” in Recent Advances in Biomagnetism, eds Yoshimoto T. Kotani M. Kuriki S. Karibe H. Nakasato N. (Sendai: Tohoku University Press), 302–305

[B47] Rols G. Tallon-Baudry C. Girard P. Bertrand O. Bullier J. (2001). Cortical mapping of gamma oscillations in areas V1 and V4 of the macaque monkey. Vis. Neurosci. 18, 527–540 10.1017/S0952523801184038 11829299

[B48] Sannita W. G. Carozzo S. Orsini P. Domenici L. Porciatti V. Fioretto M. (2009). ‘Gamma’ band oscillatory response to chromatic stimuli in volunteers and patients with idiopathic Parkinson’s disease. Vision Res. 49, 726–734 10.1016/j.visres.2009.01.018 19232367 PMC2760460

[B49] Shapley R. Hawken M. Ringach D. L. (2003). Dynamic’s of orientation selectivity in the primary visual cortex and the importance of cortical inhibition. Neuron 38, 689–699 10.1016/S0896-6273(03)00332-5 12797955

[B50] Singh K. D. (2012). Which “neural activity” do you mean? fMRI, MEG, oscillations and neurotransmitters. Neuroimage 62, 1121–1130 10.1016/j.neuroimage.2012.01.028 22248578

[B51] Singh K. D. Barnes G. R. Hillebrand A. (2003). Group imaging of task-related changes in cortical synchronisation using nonparametric permutation testing. Neuroimage 19, 1589–1601 10.1016/S1053-8119(03)00249-0 12948714

[B52] Smith S. M. (2002). Fast robust automated brain extraction. Hum. Brain Mapp. 17, 143–155 10.1002/hbm.10062 12391568 PMC6871816

[B53] Solomon S. G. Lennie P. (2005). Chromatic gain controls in visual cortical neurons. J. Neurosci. 25, 4779–4792 10.1523/JNEUROSCI.3921-04.2005 15888653 PMC6724777

[B54] Swettenham J. B. Muthukumaraswamy S. D. Singh K. D. (2009). Spectral properties of induced and evoked gamma oscillations in human early visual cortex to moving and stationary stimuli. J. Neurophysiol. 102, 1241–1253 10.1152/jn.91044.2008 19515947

[B55] Vrba J. Robinson S. E. (2001). Signal processing in magnetoencephalography. Methods 25, 249–271 10.1006/meth.2001.1238 11812209

[B56] Wachtler T. Sejnowski T. J. Albright T. D. (2003). Representation of color stimuli in awake macaque primary visual cortex. Neuron 37, 681–691 10.1016/S0896-6273(03)00035-7 12597864 PMC2948212

[B57] Woolrich M. W. Behrens T. E. J. Beckmann C. F. Jenkinson M. Smith S. M. (2004). Multilevel linear modelling for FMRI group analysis using Bayesian inference. Neuroimage 21, 1732–1747 10.1016/j.neuroimage.2003.12.024 15050594

[B58] Wyart V. Tallon-Baudry C. (2008). Neural dissociation between visual awareness and spatial attention. J. Neurosci. 28, 2667–2679 10.1523/JNEUROSCI.4748-07.2008 18322110 PMC6671201

[B59] Zumer J. M. Brookes M. J. Stevenson C. M. Francis S. T. Morris P. G. (2010). Relating BOLD fMRI and neural oscillations through convolution and optimal linear weighting. Neuroimage 49, 1479–1489 10.1016/j.neuroimage.2009.09.020 19778617

